# A comparison of vas occlusion techniques: cautery more effective than ligation and excision with fascial interposition

**DOI:** 10.1186/1471-2490-4-12

**Published:** 2004-10-27

**Authors:** David Sokal, Belinda Irsula, Mario Chen-Mok, Michel Labrecque, Mark A Barone

**Affiliations:** 1 Family Health International, PO Box 13950, Research Triangle Park, NC 27709, USA; 2 Department of Family Medicine, Laval University, Quebec City, Canada; 3 EngenderHealth, 440 Ninth Avenue, New York, NY 10001, USA

## Abstract

**Background:**

Vasectomy techniques have been the subject of relatively few rigorous studies. The objective of this analysis was to compare the effectiveness of two techniques for vas occlusion: intraluminal cautery versus ligation and excision with fascial interposition. More specifically, we aimed to compare early failure rates, sperm concentrations, and time to success between the two techniques.

**Methods:**

We compared semen analysis data from men following vasectomy using two occlusion techniques. Data on intraluminal cautery came from a prospective observational study conducted at four sites. Data on ligation and excision with fascial interposition came from a multicenter randomized controlled trial that evaluated the efficacy of ligation and excision with versus without fascial interposition. The surgical techniques used in the fascial interposition study were standardized. The surgeons in the cautery study used their customary techniques, which varied among sites in terms of type of cautery, use of fascial interposition, excision of a short segment of the vas, and use of an open-ended technique. Men in both studies had semen analyses two weeks after vasectomy and then approximately every four weeks. The two outcome measures for the analyses presented here are (a) time to success, defined as severe oligozoospermia, or <100,000 sperm/mL in two consecutive semen analyses; and (b) early vasectomy failure, defined as >10 million sperm/mL at week 12 or later.

**Results:**

Vasectomy with cautery was associated with a significantly more rapid progression to severe oligozoospermia and with significantly fewer early failures (1% versus 5%).

**Conclusion:**

The use of cautery improves vasectomy outcomes. Limitations of this comparison include (a) the variety of surgical techniques in the cautery study and differences in methods of fascial interposition between the two studies, (b) the uncertain correlation between sperm concentrations after vasectomy and the risk of pregnancy, and (c) the use of historical controls and different study sites.

## Background

Vasectomy techniques have been the subject of relatively few rigorous studies. The Royal College of Obstetricians and Gynecologists [1] noted that more research is needed to compare different methods of vasectomy. Experts have recommended the use of fascial interposition or cautery [1], cautery with clips [2], and cautery with fascial interposition [2,3].

We have shown in a randomized controlled trial that fascial interposition can reduce failure rates by about half when the occlusion method is suture ligation with vas excision [4]. However, the failure rates defined by semen analyses in that study were relatively high, with a failure rate of 5.7% even in the fascial interposition group.

At an experts' meeting organized by Family Health International (FHI) and EngenderHealth in April 2001, experts reviewed data on vas occlusion techniques, including the preliminary pooled results from the randomized controlled trial of fascial interposition mentioned above. Given the apparently high rate of vasectomy failures in that study, the experts recommended that an observational study be done to assess sequential sperm concentrations after vasectomies with cautery to see if there was a qualitative difference between sperm concentrations after vas occlusion by cautery and sperm concentrations after occlusion by ligation and excision. Some participants suggested that if such a study showed a clear difference in the rate of success or the frequency of apparent recanalizations between cautery and ligation and excision with fascial interposition, then this might provide sufficient evidence for service providers to consider switching to the use of a cautery technique [5].

Based on that recommendation, we conducted an observational study of vasectomy by cautery [6] and then conducted this comparative analysis. Even if some researchers decide that additional research is needed, the data from this comparative analysis should provide a strong basis for planning further research. The objectives of the analysis presented here were to compare early failure rates, sperm concentrations, and time to success for vas occlusion by ligation and excision with fascial interposition versus those for vas occlusion by cautery.

## Methods

The methods of the fascial interposition and cautery studies have been previously described [4,6]. In brief, the fascial interposition study involved eight sites in seven countries. It was a randomized controlled trial comparing two occlusion techniques: ligation and excision with versus without fascial interposition. All surgeons used the no-scalpel approach to the vas and a standardized occlusion technique. The vas was occluded using two silk sutures, and an approximately 1-cm segment of vas between the ligatures was excised. For the fascial interposition technique, a suture was used to contain the testicular end of the vas inside the fascial sheath; the prostatic end remained outside [4]. The study was halted following a planned interim analysis that demonstrated a clear benefit from the use of fascial interposition [7]. Of 419 men who had fascial interposition in that study, 410 were included in this comparative analysis. Nine men were excluded because of lack of follow-up data.

The cautery study involved four sites in four countries. It was designed to estimate the effectiveness of cautery as currently performed and to describe the trends in sperm counts after vas occlusion by cautery. The surgeons used their customary cautery occlusion techniques, which differed among the sites. At two sites, surgeons used electro-cautery without fascial interposition: one with and one without excision of a short segment of the vas. At the other two sites, they used thermal cautery with fascial interposition: one with and one without an open-ended technique and excision of a short segment of the vas. Three sites used the no-scalpel approach to the vas. Graphic depictions of the four methods used have been published [6]. Of 400 men enrolled, 389 are included in this comparative analysis. Eleven men were excluded because of lack of follow-up data.

### Follow-up and semen analysis methods

Both studies included frequent semen analyses, beginning at two weeks after vasectomy. In the fascial interposition study, subsequent semen analyses were conducted every four weeks until a man had provided two consecutive azoospermic specimens, was declared a vasectomy failure, or reached the end of study follow-up at 34 weeks. In the cautery study, after the first sample at two weeks, subsequent semen analyses were conducted at weeks 5, 8, 12, 16, 20, and 24, regardless of semen analysis findings. In both studies, participants were asked to record all ejaculations between semen analyses on a wallet-sized card, which they gave to study personnel at each follow-up visit.

Semen analyses methods for both studies were based on World Health Organization recommendations, but the methods differed somewhat between the two studies. Freshly collected semen was examined in the fascial interposition study, and data were obtained on sperm concentration, motility, and viability. For the cautery study, two of the four sites did not routinely collect fresh specimens, so semen analysis data from those two sites were limited to sperm concentrations. Therefore, for this comparative analysis, we did not consider sperm motility as an outcome measure. In addition, specimens showing azoospermia or very low sperm concentrations were centrifuged in the fascial interposition study but not in the cautery study. During both studies, the laboratories conducted periodic quality-control tests.

### Outcome measures

In both studies, we used frequent semen analyses rather than pregnancy as the vasectomy effectiveness outcome measure, to minimize the risk of pregnancy, sample size, and study duration. Vasectomy success is commonly defined as two azoospermic specimens [2]. However, small numbers of nonmotile sperm may persist for many months in some men. Surgeons' experience [8] and guidelines recently published by the British Andrology Society [9] suggest that low concentrations of nonmotile sperm (<100,000 sperm/mL) are of less concern than higher concentrations. We found in the fascial interposition study [4] that severe oligozoospermia (<100,000 sperm/mL) was a more robust measure of success than was azoospermia, at least for research purposes. In both studies, men of different ages tended to reach severe oligozoospermia at about the same time, but older men took longer to reach azoospermia than did younger men.

Consequently, we used two definitions for vasectomy success for this comparative analysis. Our primary definition of success was severe oligozoospermia, defined as <100,000 sperm/mL in two consecutive specimens taken at least two weeks apart. Our alternate definition of success was the occurrence of two consecutive azoospermic specimens taken at least two weeks apart, with no subsequent samples showing sperm concentrations of 100,000 sperm/mL or higher. Motility was not considered, for reasons mentioned earlier. The date of success was the date of the first of the two oligozoospermic or azoospermic semen samples.

For early failure, we used a criterion of >10 million sperm/mL at week 12 or later, regardless of motility. This is an adaptation of Alderman's criteria specifying 5 million motile sperm/mL or more as evidence of "overt failure" [10]. This definition is different from the definitions of failure used by each of the two studies, but it was necessary for a comparative analysis because some sites in the cautery study did not measure sperm motility. Thus, the failure rates reported here may differ slightly from the failure rates reported by each study. In addition, to avoid bias from the two studies' different lengths of follow-up, we included semen analysis data from the fascial interposition study through only 26 weeks of follow-up.

The data collection forms, study monitoring, and laboratory quality-control procedures were similar for both studies, though only one research site was common to both. Both studies were organized and managed by researchers and staff at FHI and EngenderHealth, and both received approval from FHI's institutional review board and from institutional review boards at the study sites.

### Statistical methods

Kaplan-Meier product-limit estimates of the probabilities of severe oligozoospermia, at each scheduled week of follow-up through week 24, and their 95% confidence intervals (CIs) were produced overall, by study group (i.e., fascial interposition and cautery), and by study group and age group (i.e., <35 years and 35 years and older). Peto's standard error [11] was used to compute the 95% CIs. The Kaplan-Meier probabilities were compared between the study groups using a two-sided log-rank test with an alpha of 0.05. Given that the participants in the fascial interposition study had a longer follow-up period than the participants in the cautery study (34 versus 24 weeks), the information on the fascial interposition participants was censored at the 26-week visit for the purpose of this comparative study.

The comparison of failure rates between the two study groups was based on a Fisher exact test with a two-sided alternative hypothesis and an alpha of 0.05. In addition, a logistic model was fit to estimate an age-adjusted odds ratio of the failure rates and its 95% CI.

Unlike in the cautery study, participants in the fascial interposition study were discontinued after azoospermia was confirmed or after vasectomy failure was declared. Therefore, for the purpose of comparing the distribution of the participants in the different sperm concentration categories by week of follow-up, we kept azoospermic cases in the azoospermic category for all follow-up weeks after their discontinuation due to confirmed azoospermia. Similarly, we kept participants with a declared vasectomy failure in the sperm concentration category that they were in at the moment of discontinuation, for all subsequent follow-up weeks.

## Results

Detailed results for the two studies have been reported [4,6]. We report here the results of the comparison of the semen analysis data from the two studies.

### Baseline population data

Among the baseline population characteristics (Table 1), age distribution was somewhat different between the two studies, with a younger population in the fascial interposition study. Marital status, number of children, use of condoms, and years of education were similar between the two studies.

### Analysis of early failures

We found significantly fewer early failures in the cautery study than in the fascial interposition group from the randomized controlled trial: 1.0% (4/389) versus 4.9% (20/410) (p = 0.0014 by the Fisher exact test). The adjusted odds ratio was 4.8 (95% CI, 1.6–14.3), indicating nearly a five-fold higher risk of early failure in the fascial interposition study than in the cautery study. No significant age effect was detected (data not shown).

### Sperm concentrations

**Figure 1:**
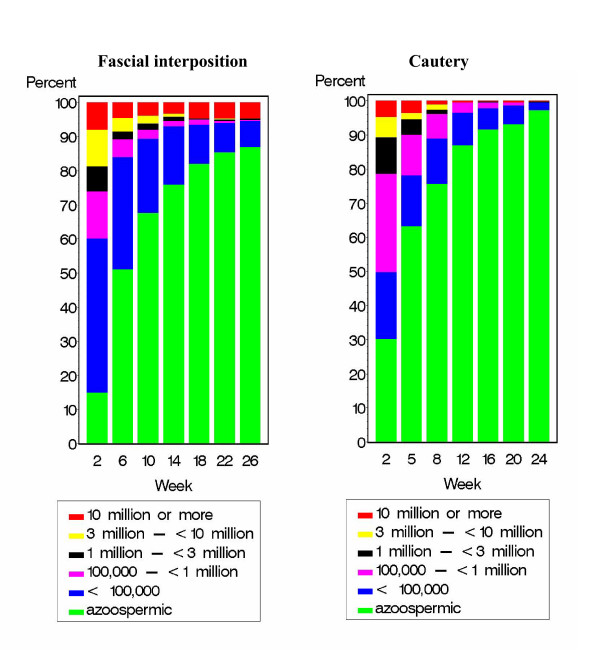
Distribution of sperm concentrations by week after vas occlusion by ligation and excision with fascial interposition (left) versus cautery (right).

The distribution of sperm concentrations by week is shown for the two studies (Figure 1). The difference in early failures can be appreciated by examining the percentages of men with high sperm concentrations. In the fascial interposition study, the percentage of men with sperm counts of 10 million or more stayed about the same from 6 to 26 weeks. However, in the cautery study, the percentage decreased dramatically from 5 to 8 to 12 weeks. This difference was probably due to recanalizations, which become apparent in the first 6 to 10 weeks after the procedure.

### Time to success

**Figure 2:**
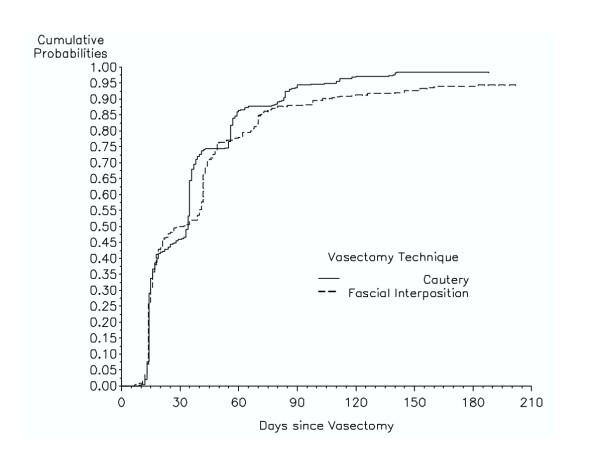
Cumulative probability of vasectomy success by week: vas occlusion by cautery (solid line) versus fascial interposition (dashed line). Success was defined as severe oligozoospermia (i.e., <100,000 sperm/mL).

Life-table analyses of time to success showed that the participants in the cautery study reached severe oligozoospermia significantly more rapidly than did those in the fascial interposition study (p = 0.0049) (Figure 2). Ninety-seven percent of the men in the cautery study had reached success by 12 weeks, while only 91% in the fascial interposition study had reached success by 14 weeks. The analyses of the data stratified by age group showed similar results (data not shown). Using the time to azoospermia outcome, the difference between the two groups was also significant (p < 0.0001) (data not shown).

## Discussion

The difference in the observed failure rates suggests that vas occlusion techniques that include cautery are significantly more effective than ligation and excision plus fascial interposition, at least based on semen analysis. We believe that most of the failures in the fascial interposition group were due to early recanalizations within the first two to three months after vasectomy (data not shown). Possible explanations for the surprisingly high failure rate among the fascial interposition group have been previously presented and discussed [4].

Recent reports suggest that pregnancy rates may be higher in low-resource settings, where semen analysis is usually not available and where most vasectomies are done by ligation and excision [12,13]. The use of cautery devices may have the potential to reduce failure rates in low-resource settings.

### Which cautery technique is best?

When a ligation and excision technique is used, we have shown that fascial interposition provides an important improvement in effectiveness [4]. The addition of fascial interposition may be less important when cautery is used as the occlusion method, but this study was not designed to answer that question.

Schmidt was a pioneer in the use of cautery for vasectomy. His preferred technique was thermal cautery of about 5 mm of each end of the vas, combined with fascial interposition and no excision [3]. Two of the sites in the observational cautery study used techniques very similar to Schmidt's technique. The other two sites used electro-cautery without fascial interposition, an approach that has been used for many years at the Elliot-Smith Clinic in Oxford [14] and similar to one used for many years by Marie Stopes clinics with excellent results [15]. Little evidence is available to support one type of cautery over another. Schmidt [16] preferred thermal cautery to electro-cautery based on histological examination of specimens at vaso-vasostomy, and Li [17] found a lower failure rate with thermal cautery than with electro-cautery, but the difference in Li's study was not statistically significant.

The length of the vas segment that is cauterized can vary by the type of cautery. In the United States and Canada, marketed thermal cautery "vasectomy tips" have a functional length of about 0.8 cm, so it would be difficult to cauterize more than 1 cm of each end. The cautery tip of the Sturgeon cautery device used by Schmidt was 0.5 cm long. However, the tips available for electro-cautery devices are much longer, which could permit cauterization of longer segments of the vas and potentially cause more difficulty in the event of a request for reversal.

Since several reports suggest that the combination of thermal cautery plus fascial interposition is one of the most effective methods available [18], this procedure can be recommended with few reservations. However, there is at least one other reason to consider the inclusion of fascial interposition in the vasectomy procedure, especially in low-resource settings. If providers in low-resource settings adopted a cautery technique, cautery instruments could occasionally become unavailable for various reasons. In those cases, a provider might want to be able to perform fascial interposition as part of a ligation and excision procedure.

Schmidt suggested that cautery should not be combined with suture or clip ligation of the vas [3]. He noted that while blood vessels will thrombose after ligation, the vas will remain open. Thus, using a ligature in addition to cautery could reduce the value of cautery by causing necrosis of some or the entire cauterized end, potentially reducing the benefit from cautery. None of the cautery techniques in this study included the use of ligatures or clips on top of a cauterized vas.

### Study limitations

Limitations of this comparison include (a) the variety of vas occlusion techniques used in the cautery study and differences in methods of fascial interposition between the two studies, (b) the uncertain correlation between post-vasectomy sperm concentrations and the risk of pregnancy, (c) the lack of sperm motility data and centrifugation for the cautery study, and (d) the use of different study sites and surgeons in the two studies. The difference in time to vasectomy success could in part be related to the lack of centrifugation in the cautery study. However, the difference in centrifugation between the two studies would not have affected the detection of early failures, especially since the cautery study gathered semen samples throughout the 24-week follow-up period (i.e., men continued providing semen samples even after they reached azoospermia). Another potential limitation of this comparison was the difference in follow-up schedules between the two studies.

In addition, other factors could have an unknown impact on the comparability of the data. Even though the results are encouraging for the use of cautery in vasectomy, we have to be cautious about making definitive statements based on this nonrandomized comparison.

### Future vasectomy research

Results from this comparative analysis suggest that cautery may be a more robust and less technique-dependent method than is fascial interposition. However, additional research would be useful to directly compare the effectiveness of the following standardized techniques in a randomized controlled trial: cautery without fascial interposition, cautery with fascial interposition, and ligation and excision with fascial interposition. Additional research would also be of interest to compare an open-ended procedure with a closed-end procedure. Several investigators have suggested that leaving the testicular end open reduces post-vasectomy pain, but no randomized controlled trials have examined this issue [1,18].

## Conclusions

We compared data from two prospective multicenter studies conducted using similar methodologies. We found that the use of cautery as part of the vasectomy procedure significantly reduced vasectomy failure rates compared with ligation and excision plus fascial interposition as part of the procedure. It is unclear from our results and those of others whether fascial interposition used with cautery improves vasectomy success rates when compared with cautery alone.

## Competing interests

The authors declare that they have no competing interests.

## Authors' contributions

DS participated in the conception, design and analysis of the study, and drafted the manuscript. BI participated in the conception, design, and analysis of the study, and was primarily responsible for managing the study implementation. MC participated in the design of the study and was primarily responsible for the statistical analysis. ML participated in the conception, design and analysis of the study. MB participated in the conception, design, management and analysis. All authors reviewed and approved the final manuscript.

## Pre-publication history

The pre-publication history for this paper can be accessed here:



## Figures and Tables

**Table 1:** Baseline characteristics by study/vas occlusion technique

**Characteristic**		**Ligation and excision with fascial interposition**		**Intraluminal cautery**	
		**N**	**%**	**N**	**%**

**Total**		410	100.0	389	100.0
**Age group**	<30	109	26.6	39	10.0
	30–34	125	30.5	115	29.6
	35–39	95	23.2	121	31.1
	40+	81	19.8	114	29.3
**Marital status**	Married	344	83.9	276	71.0
	Co-habiting	52	12.7	92	23.7
	Single	14	3.4	21	5.4
**Number of children**	0	20	4.9	16	4.1
	1	22	5.4	44	11.3
	2	210	51.2	196	50.4
	3+	158	38.5	133	34.2
**Prior condom use**	No	213	52.0	178	45.8
	Yes	197	48.0	211	54.2
Years of education *	Mean (SD)	11.0 (4.3)		13.1 (3.7)	
	Median	11		13	

* One "years of education" value was missing for the fascial interposition group.
